# Supplementary material for: Histone Acetylation Domains Are Differentially Induced during Development of Heart Failure in Dahl Salt-Sensitive Rats
Source: Int J Mol Sci. 2021 Feb 10;22(4):1771. doi: 10.3390/ijms22041771 (PMC7916721; doi:10.3390/ijms22041771)
Supplement: Supplementary file 1 [file ijms-22-01771-s001.pdf]

Supplemental Figure. 1

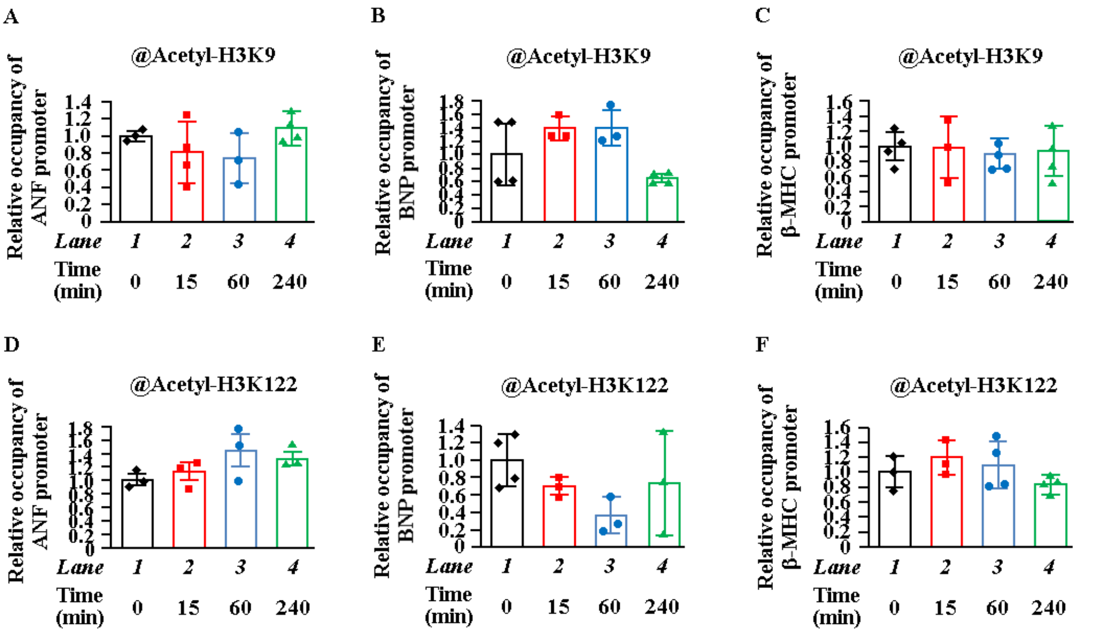

Supplemental Fig. 1 The acetylation levels of H3K9 and H3K122 were not changed around the upstream region of the hypertrophic response gene promoters

(A-F) ChIP assays were performed using cardiomyocyte lysates treated with or without PE for 0, 15, 60, or 240 min with anti-acetyl-histone H3K9 antibody (A-C), anti-acetyl-histone H3K122 antibody (D-F), or normal rabbit IgG as a negative control (not detected). N=3 to 4; *one-way* ANOVA followed by Tukey test. * $p < 0.05$.

Supplemental Figure. 2

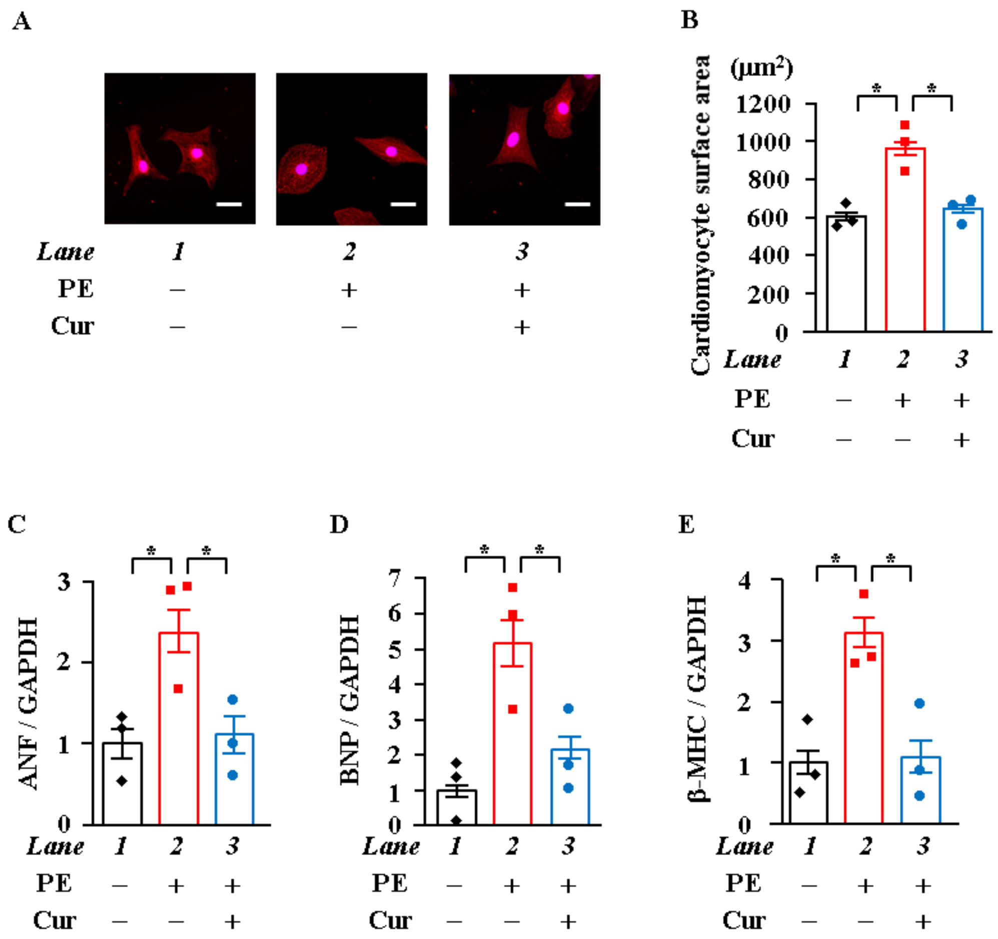

Supplemental Fig. 2 Curcumin treatment inhibited cardiomyocyte hypertrophy

Primary cultured neonatal rat cardiomyocytes were treated with curcumin (10 μM) for 2 hours, then with PE (30 μM), as described in Fig. 2. **(A and B)** Immunofluorescence staining was performed with anti-MHC antibody. The areas of 50 randomly-chosen cells were measured using ImageJ v4.16. (A) is representative photographic image of cardiomyocytes, and (B) is a quantification of (A). Scale bar: 20 μm. **(C-E)** mRNAs were extracted from the cardiomyocytes, and mRNA levels of (C) ANF, (D) BNP, and (E) β-MHC were measured by qRT-PCR assay. **(B-E)**, N=3; *one-way* ANOVA followed by Tukey test. * $p < 0.05$

Supplemental Figure. 3

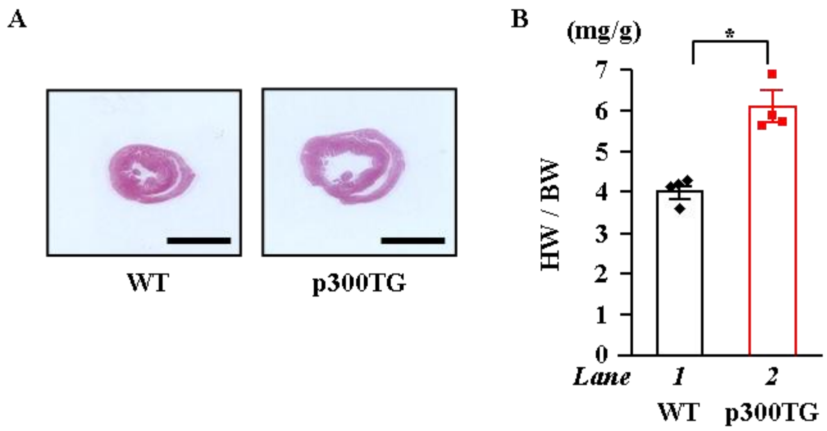

Supplemental Fig. 3 Cardiac hypertrophy was observed in TG mice with cardiac overexpression of p300

(A) The hearts of p300-TG mice were subjected to hematoxylin eosin staining at 26 weeks of age. Scale bar: 5 mm. **(B)** Heart weight / body weight (HW/BW) ratios at 26 weeks of age. (**B**), N=4; unpaired t-test. * $p < 0.05$

Supplemental Table 1

Stage	LVH		HF	
**Parameter**	**DR 12w**	**DS 12w**	**DR 21w**	**DS 21w**
**LVPWd (mm)**	**2.2 ± 0.3**	**3.2 ± 0.3***	**2.4 ± 0.2**	**3.4 ± 0.3†**
**IVSd (mm)**	**1.9 ± 0.2**	**2.6 ± 0.3***	**1.8 ± 0.3**	**3.0 ± 0.3†‡**
**LVIDd (mm)**	**6.3 ± 0.4**	**5.7 ± 0.6**	**7.1 ± 0.2**	**6.9 ± 0.5**
**IVIDs (mm)**	**2.7 ± 0.4**	**2.2 ± 0.6**	**3.1 ± 0.2**	**4.6 ± 0.4†‡**
**FS (%)**	**57.8 ± 5.5**	**62.9 ± 6.9**	**57.1 ± 4.0**	**34.2 ± 5.2†‡**

Supplemental Table 1. The data of echocardiography from salt resistant and sensitive Dahl rats
Abbreviations: LVH, Left ventricular hypertrophy; HF, Heart failure; DR, Dahl salt-resistant rat; DS, Dahl salt-sensitive rat; LVPWT, Left ventricular posterior wall thickness; IVSd, Interventricular septum thickness at end-diastole; LVIDd, left ventricular internal diameter at end diastole; IVIDs, left ventricular internal diameter at end systole; FS, Fractional shortening. N=4; two-*way* ANOVA followed by Tukey test. * $p < 0.05$ DR 12w vs DS 12w, † $p < 0.05$ DR 21w vs DS 21w, ‡ $p < 0.05$ DS 12w vs DS 21w
